# Mapping biological influences on the human plasma proteome beyond the genome

**DOI:** 10.1038/s42255-024-01133-5

**Published:** 2024-09-26

**Authors:** Julia Carrasco-Zanini, Eleanor Wheeler, Burulça Uluvar, Nicola Kerrison, Mine Koprulu, Nicholas J. Wareham, Maik Pietzner, Claudia Langenberg

**Affiliations:** 1 Precision Healthcare University Research Institute, Queen Mary University of London, London, UK; 2 Computational Medicine, Berlin Institute of Health at Charité-Universitätsmedizin Berlin, Berlin, Germany; 3 MRC Epidemiology Unit, University of Cambridge School of Clinical Medicine, Institute of Metabolic Science, Cambridge, UK

**Keywords:** Proteomic analysis, Epidemiology, Biomarkers, Metabolism, Proteins

## Abstract

Broad-capture proteomic platforms now enable simultaneous assessment of thousands of plasma proteins, but most of these are not actively secreted and their origins are largely unknown. Here we integrate genomic with deep phenomic information to identify modifiable and non-modifiable factors associated with 4,775 plasma proteins in ~8,000 mostly healthy individuals. We create a data-driven map of biological influences on the human plasma proteome and demonstrate segregation of proteins into clusters based on major explanatory factors. For over a third (*N* = 1,575) of protein targets, joint genetic and non-genetic factors explain 10–77% of the variation in plasma (median 19.88%, interquartile range 14.01–31.09%), independent of technical factors (median 2.48%, interquartile range 0.78–6.41%). Together with genetically anchored causal inference methods, our map highlights potential causal associations between modifiable risk factors and plasma proteins for hundreds of protein–disease associations, for example, COL6A3, which possibly mediates the association between reduced kidney function and cardiovascular disease. We provide a map of biological and technical influences on the human plasma proteome to help contextualize findings from proteomic studies.

## Abstract (web summary)

The authors systematically study biological influences on the human plasma proteome in a large cohort, thereby revealing causal associations between plasma proteins and modifiable risk factors for protein–disease associations.

## Main

Proteins are the main effector molecules on cellular function and represent the largest class of drug targets^1^ but have been largely inaccessible at scale in human studies. Broad-capture profiling platforms can now measure thousands of proteins in blood putting humans back at the centre of basic and translational research. However, only about ~10% of the proteins measured are predicted to be actively secreted into the blood^2^ leaving the origin and role of most plasma proteins unclear. Previous work largely studied underlying determinants in isolation, including the contribution of DNA sequence variation, so-called protein quantitative trait loci (pQTLs)^3–5^, body fat^6^, liver and kidney function^7^, modifiable lifestyle factors^8^ and the gut microbiome^9^, or a large body of work on technical factors^10^. While these studies revealed strong contributions of specific factors, explaining more than 10% of the variation in selected protein targets^11^, they fell short in deciphering the multifactorial contribution of diverse organ systems responding to endogenous and exogenous stimuli determining blood protein levels.

Such knowledge, however, is critical to contextualize the rapidly increasing number of proteomics studies ranging from small clinical cohorts of rare diseases to large prospective studies investigating common non-communicable diseases. For example, identifying modifiable risk factors for proteins known to play a causal role in disease aetiology can help developing cost-effective treatment strategies with minimal adverse effects and that might synergistically improve effectiveness of pharmaceutical compounds.

In this article, we systematically characterized the contribution of diverse modifiable risk factors, genetic determinants and technical factors to aptamer-based plasma abundance measurements of 4,775 distinct proteins. We illustrate how the identification of potentially causal modifiable determinants can improve the biological interpretation of disease-associated proteins and inform on modifiable risk factors that have the potential to modulate disease mechanisms.

## Results

We used an aptamer-based assay to measure 4,775 plasma protein targets (targeted by 4,979 aptamers) in 8,350 participants from the Fenland study (Supplementary Table 1). We integrated detailed information on participant characteristics, including genetic, dietary, lifestyle and health information, objective measurements of physical activity, and anthropometric and blood-based clinical biomarker measurements (Methods).

### Major biological factors associated with the plasma proteome

**Fig. 1:**
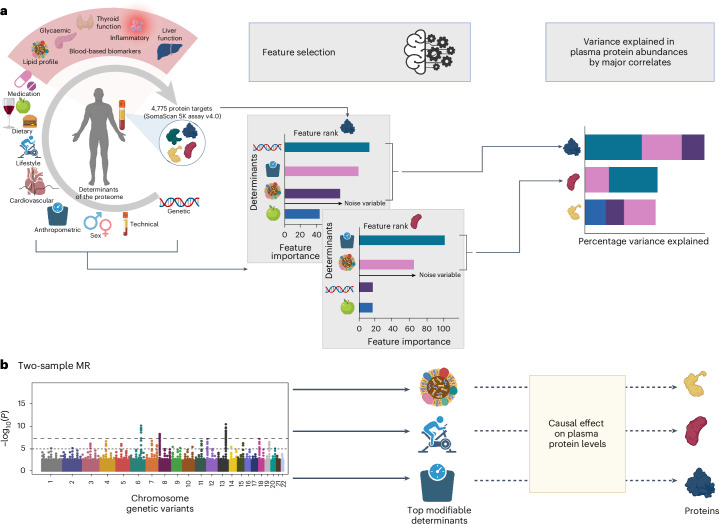
Scheme of the study design. **a**, We performed a feature selection step to identify relevant explanatory variables for each protein, while avoiding selection of highly correlated variables. These pre-selected sets of features were then used to fit linear mixed models for each protein to quantify the proportion of variance in plasma abundance attributable to each explanatory feature. **b**, We performed a two-sample MR to test whether major explanatory factor are causally associate to their corresponding proteins. Variants were selected based on genome-wide significance (*P* < 5 × 10^−^^8^; dashed lines). Created with BioRender.com.

**Extended Data Fig. 1:**
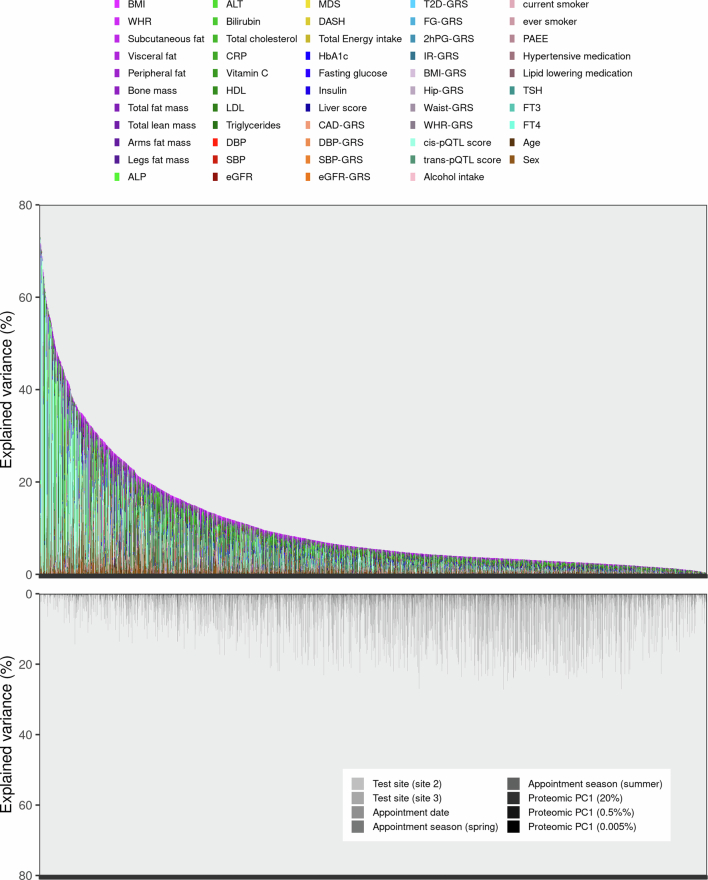
Participant characteristics and genetic factors explaining the variation in plasma proteins. Total variance explained in plasma abundances of each protein target by a range of phenotypic and genetic determinants (upper panel) and technical factors (lower panel).

We identified between 4 and 56 (median 25) characteristics (Supplementary Table 2) as important explanatory factors for each protein target using a machine-learning approach to preselect highly informative but largely uncorrelated variables (Methods and Fig. 1). For more than half of the protein targets (*n* = 2,841 aptamers; 57%, 2,759 unique proteins), technical variation was the largest contributor to the variation in plasma abundance, explaining a median of 7.29% (interquartile range (IQR) 3.36–13.03%) (Extended Data Fig. 1 and Supplementary Table 3). To enhance biological interpretation, we therefore regressed out technical factors for all downstream analyses and identified the major biological factors associated with plasma levels for each of the protein targets.

**Fig. 2:**
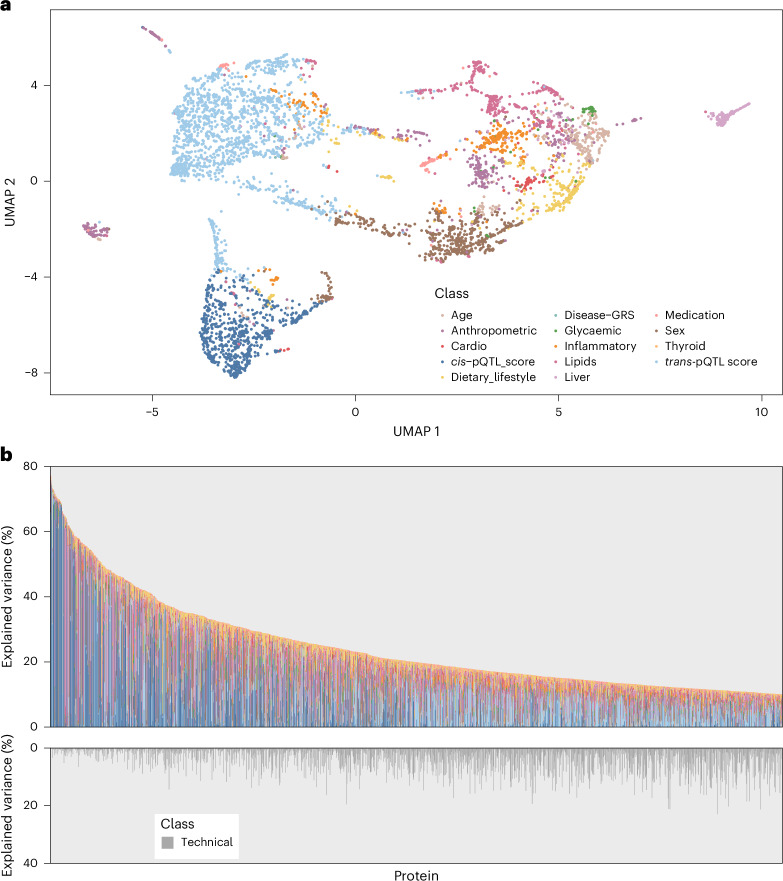
Contribution of modifiable participant characteristics or genetic factors to the variation in plasma abundances of the proteome. **a**, UMAP projection of the matrix of variance explained for all protein targets. Proteins are coloured according to the class of the major contributor to their variation in plasma. GRS, genetic risk score. **b**, The total variance explained in the plasma abundance of each protein target by a range of participant characteristics and genetic factors, which are coloured according to higher order categories. We only show protein targets for which more than 10% of the variation in plasma was explained.

We observed a segregation of plasma proteins into distinct clusters depending on the major biological explanatory factors (those that explained the largest proportion of variance for each protein target) when projecting the variance-explained matrix (Supplementary Table 4) using uniform manifold approximation and projection (UMAP) into a two-dimensional space (Fig. 2a). UMAP is a dimensionality reduction technique that optimizes a low-dimensional graph to be as similar as possible to a high-dimensional graph representation of the data (that is, the variance-explained matrix). Annotating protein targets with their major associated factor (highest explained variance) indicated that the spatial separation in UMAP space was mainly driven by these, forming clear clusters. Most proteins were best explained by non-modifiable factors, that is, genetic variation, age or sex (*n* = 3,242 protein targets), although a considerable number (*n* = 1,737) appeared to be best explained by modifiable factors (Supplementary Table 5).

Participant characteristics and genetic factors jointly explained a median of 5.49% (IQR 3.00–13.8%) of variation in plasma protein abundance. For one-third of the protein targets (*n* = 1,637 aptamers; 1,575 unique proteins) these explained more than 10% of the variation (median 19.88%, IQR: 14.01% to 31.09%), independent of the influence of technical factors (median 2.48%, IQR: 0.78% to 6.41%) (Fig. 2b). We observed values as high as 77.3% for neurexin 1 (NRX1A), mainly explained by the *trans*-pQTL score, 70.02% for matching clinical chemistry assays (such as the C-reactive protein (CRP)-targeting aptamer), but also up to 64.60% and 58.58% for aminoacylase-1 and matrix-associated remodelling associated protein 8, explained by a range of different participant characteristics and genetic factors (Supplementary Table 5). We make our results available through an interactive web server across all proteins and explanatory variables tested (https://omicscience.org/apps/protdeterminants).

While sex was on average the most important non-modifiable phenotypic factor associated (median 0.55%, up to 60.22%), two-thirds of the protein targets were mostly explained by genetic factors, with variation within or near to the protein coding genes (*cis*-pQTLs; median variance explained of 3.10%, up to 74.27%) explaining more than the aggregated effects elsewhere in the genome (*trans*-pQTL score; median 1.35%, up to 71.98%). The still considerable amount of variation by *trans*-pQTLs is probably explained by the effects on protein–protein interaction partners, protein complexes or the cumulative effect of pathways regulating the target protein, but might also be due to unspecific mechanisms related to the measurement technique or sample handling as outlined prevoiusly^4,12,13^. Genome-wide single-nucleotide polymorphism (SNP)-based heritability estimates were broadly correlated with the total proportion of variance explained by the *cis*- and *trans*-pQTL scores (Pearson *r* = 0.73; 95% confidence interval (CI) 0.71 to 0.74); Supplementary Table 6).

**Fig. 3:**
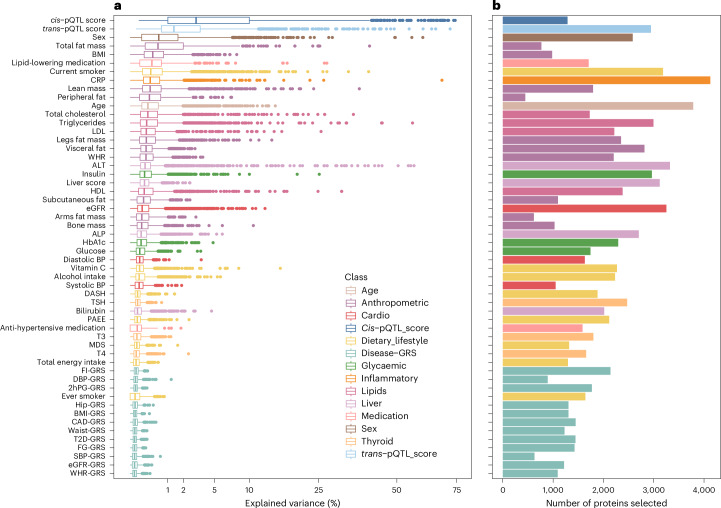
Summary of explanatory variable selection and overall contribution to the variation in the plasma proteome. **a**, The distribution of variance attributable to each explanatory variable across all proteins for which they were selected (*N* = 447–4,125). The *x* axis has been square root transformed to improve visibility. The box plot depicts the median, IQR, first quartile − 1.5 × IQR, and third quartile + 1.5 × IQR. The points represent outliers. WHR, waist-to-hip ratio; eGFR, estimated glomerular filtration rate; SBP, systolic blood pressure; FG, fasting glucose; CAD, coronary artery disease; GRS, genetic risk score; BMI, body mass index; 2hPG, 2 hour plasma glucose following an oral glucose tolerance test; DBP, diastolic blood pressure; FI, fasting insulin; T4, thyroxine; T3, triiodothyronine; MDS, Mediterranean diet score; PAEE, physical activity energy expenditure; TSH, thyrotropin; DASH, Dietary Approaches to Stop Hypertension diet score; ALP, alkaline phosphatase; HDL, high-density lipoprotein cholesterol; ALT, alanine aminotransferase; LDL, low-density lipoprotein cholesterol; CRP, C-reactive protein. **b**, The total number of proteins for which explanatory factors were selected and therefore included in the linear mixed models.

In contrast, putative modifiable factors such as chronic low-grade inflammation (CRP explaining up to 68.34% of variation), liver function (alanine transaminase (ALT) explaining up to 56.66% of variation), kidney function (estimated glomerular filtration rate (eGFR) explaining up to 12.79% of variation), and current smoking status (explaining up to 39.98% of variation) explained variation in plasma levels of most proteins but on average explained a relatively small proportion (median variance explained between 0.10% and 0.29%). Measures of body composition, such as body mass index (BMI; median 0.36%, up to 7.99%), total fat mass (median 0.55%, up to 40.30%) or peripheral fat (median 0.26%, up to 7.23%) were informative for relatively fewer proteins but overall explained more variance for most of the protein targets, if selected (Fig. 3).

**Fig. 4:**
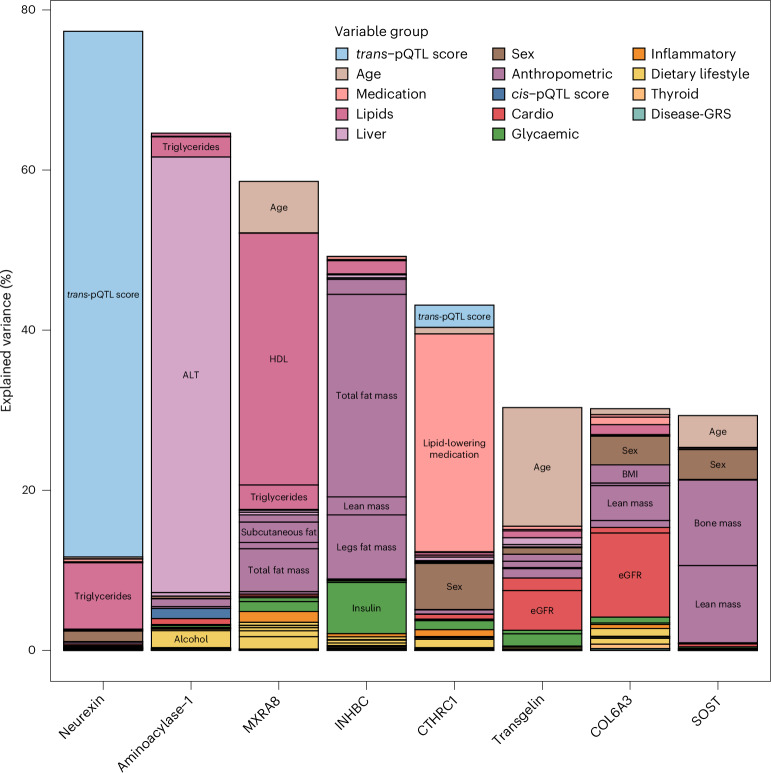
Contribution of modifiable participant characteristics or genetic factors to the variation in selected proteins’ plasma abundance. Examples of variance attributable to participant characteristics and genetic factors are shown for selected examples. GRS, genetic risk score.

Proteins that were selectively explained by few, maybe even only one, risk factor (Fig. 4) are promising biomarkers for health-related changes, for example, bone mass for plasma sclerostin (10.7%), age for transgelin (14.8%), lipid-lowering medication for collagen triple helix repeat-containing protein 1 (CTHR1 or CTHRC1, 27.2%) and procollagen C-endopeptidase enhancer 1 (26.5%), or plasma vitamin C levels and DH-cytochrome b5 reductase 3 (15.9%). The positive association (Supplementary Table 7) between lipid-lowering medication (mostly statins in the Fenland population) and CTHRC1, which was comparatively stronger compared with its association with proteins directly related to hepatic lipoprotein uptake such as PCSK9 (17.3%) might point to potential off-target effects. CTHRC1 is highly expressed in bone tissue and *Cthrc1* knockout mice are susceptible to osteoporotic fractures^14^. Accordingly post-menopausal women on statin therapy have a lower fracture risk^15^, which might suggest that a statin-induced increase in CTHRC1 could contribute to this protective effect. To follow-up on other potential off-target medication effects, we looked-up the results for anti-hypertensive medications, but these were only the major associated factors for three proteins, explaining a very small proportion of variation in plasma (0.24–0.40%). We further highlight that our statistical framework is not fully able to differentiate the effects of the medications per se from that of the underlying risk factors (for example, that of statins versus the effect of low-density lipoprotein (LDL) cholesterol).

We^12^ and others^16^ have previously shown that biological associations identified for a given protein target can differ depending on the technology used for measurement, in part influenced by each assay’s sensitivity and specificity for detecting specific isoforms of the same protein. We therefore sought to test the validity and generalizability of our results using an alternative proteomic technology (Olink’s proximity extension assay). To distinguish technology specific from any other study design and population-driven differences, analyses need to be performed and compared in the same set of individuals; we investigated the proportion of variance explained for 273 well-matched assay pairs (*r* > 0.6) in a subset of 475 participants from the Fenland study (Methods). We observed a very high correlation (Spearman *r* = 0.86) of the total proportion of variance explained between matching assays, and for 53% of those, we identified the same major biological influence on plasma abundances, explaining a similar proportion of variance (Spearman *r* = 0.80; Supplementary Table 8).

### Tissue of origin and drug targets

**Extended Data Fig. 2:**
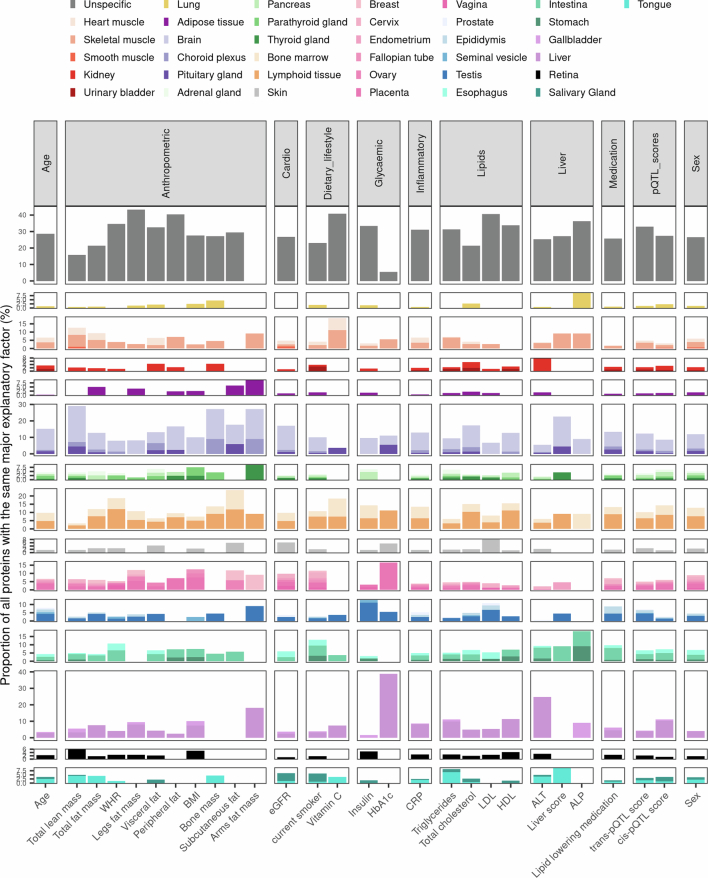
Tissue-specific or -enhanced expression among proteins influenced by the same major explanatory biological variable. Major explanatory factors are listed in the x axis. The y axis represents among all the proteins with the same major explanatory factor (listed in the x axis), what proportion is annotated to specific tissues according to the Human Protein Atlas data. Facets represent grouping by explanatory factor classes and related tissue classes.

We next tested alignment between the major associated factors of the plasma proteome and tissue-specific or enhanced expression of protein targets (Methods). For example, proteins actively secreted into plasma were enriched among those best explained by low-grade inflammation (odds ratio (OR) 2.09, 95% CI 1.21 to 3.79; pointing to active secretion of cytokines and other signalling molecules), but depleted among those best explained by liver damage (OR 0.39, 95% CI 0.13 to 1.04; suggesting cellular lysis as an underlying phenomenon leading to leakage of intracellular proteins). In general, we did not observe strong evidence of correspondence between factors strongly associated with plasma protein levels and enhanced/specific expression in tissues closely linked to the respective factor (Extended Data Fig. 2). The only exceptions were proteins specifically expressed in adipose tissue, which were often best explained by anthropometric assessment compared with other traits (Extended Data Fig. 2). Collectively, these results suggest that tissue-specific secretion or leakage is unlikely to account for our observations.

Among the assayed proteins were 586 reported drug targets^17^ for which our results could provide insights into potential treatment-modifying factors or adverse effects, assuming that plasma concentrations reflect tissue expression to some extent (Supplementary Table 9). We observed that *trans*- (*N* = 187) and *cis*-pQTLs (*N* = 81), sex (*N* = 67), and smoking status (*N* = 51) were major influences on the most druggable targets. While *cis*-pQTLs might influence target engagement or accessibility due to structural variations, the strong effect of sex on targets such as cluster of differentiation 2 (CD2; 18.7%) or toll-like receptor 5 (TLR5; 18.2%) argues for the investigation of sex-specific dosing schemes. Examples of potential adverse effects include the high amount of variance explained in plasma levels of cytochrome P450 3A4 by plasma triglyceride levels (20.4%). Cytochrome P450 3A4 is the target of small molecular inhibitors like ritonavir^18^ that are used to increase bioavailability of antiretroviral treatments for human immunodeficiency virus or coronavirus disease 2019 with increased triglyceride levels being a reported side effect^19^. In general, we hope our results can help in guiding follow-up or retrospective studies in clinical trials to tailor drugs to patient characteristics.

### From associations to determinants of the plasma proteome

**Fig. 5:**
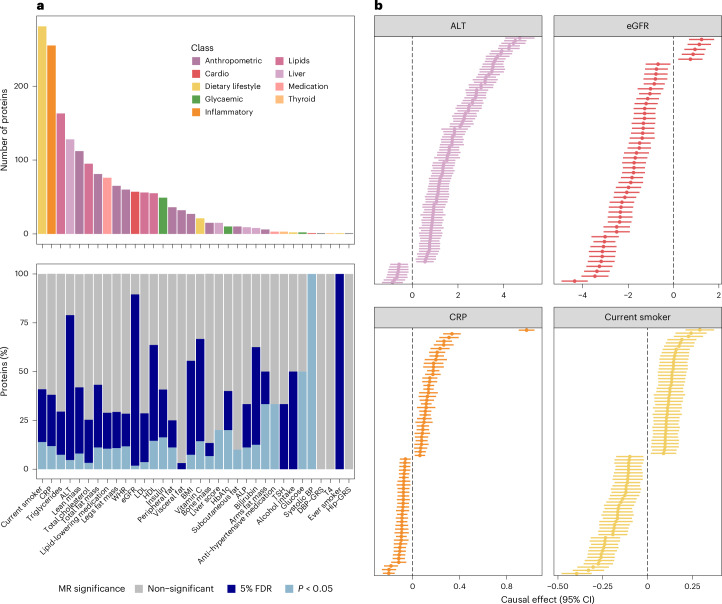
Causal determinants of the plasma proteome. **a**, Top: for each determinant, the total number of proteins for which it was the major contributor to the variance explained in their plasma levels is shown. Bottom: the proportion of proteins for which the major determinant was causally associated at a 5% FDR (dark blue) threshold or nominally (light blue) associated. **b**, Causal estimates for determinant protein associations (which were significant at a 5% FDR threshold) for ALT, eGFR, CRP and current smoking status. Each line represents a protein. The centres represent causal *β* coefficients (change in plasma protein per 1 s.d. increase of genetically determined exposure) with the 95% CIs. Dashed lines indicate null effects. CRP, C-reactive protein; ALT, alanine aminotransferase; WHR, waist-to-hip ratio; eGFR, estimated glomerular filtration rate; LDL, low-density lipoprotein cholesterol; HDL, high-density lipoprotein cholesterol; BMI, body mass index; HbA1c, glycated hemoglobin; ALP, alkaline phosphatase; TSH, thyrotropin; BP, blood pressure; DBP, diastolic blood pressure; GRS, genetic risk score; T4, thyroxine.

We next systematically identified putative causal associations among the major modifiable explanatory factors (that is, those explaining the largest proportion of variance in plasma levels) and their corresponding proteins by performing two-sample Mendelian randomization (MR) at scale (Supplementary Table 10). We identified 541 (31.15% of all associations tested) that were probably causal variable—protein pairs (at 5% false discovery rate (FDR)) of which 128 (tier 1, 7.37% of all associations tested) persisted in extensive sensitivity analysis, whereas we consider the remainder as tier 2 findings (Supplementary Table 11). Major modifiable factors were putatively causally related to up to 87.7% of their associated protein targets (median of 12 proteins causally associated; range: 1–95). Indicators of liver injury (serum ALT, 95 out of 128 proteins) and kidney function (eGFR, 50 out of 57 proteins) were genetically linked to the largest fraction of associated proteins, while current smoking status (76 out of 281) and a marker of chronic low-grade inflammation (CRP, 67 out of 255) were genetically linked to many proteins, albeit at a lower overall fraction (Fig. 5a). We note that our MR analysis considered biomarkers such as serum ALT, eGFR and CRP as proxies for systemic effects. As such, these results do not imply a direct role of either ALT, eGFR or CRP in plasma levels of the associated proteins. We rather argue that liver damage, evidenced by increased ALT (causal estimates representing the change in protein-relative units per 1 standard deviation (s.d.) increase of genetically determined exposure range from −0.87 to 4.71; 91.57% positive causal effects), and decreased kidney function, evidenced by lower eGFR (causal estimates range from −4.36 to 1.24; 90% increasing causal effects), are major determinants for increased plasma protein levels. In turn, low-grade inflammation and smoking seem to rather shift the composition of the associated plasma proteome, with effects in both directions (Fig. 5b and Supplementary Table 11). We further observed strong evidence of putatively causal associations between risk factors that were specific for few proteins such as bone mineral density for sclerostin (causal *β* = 0.20, 95% CI 0.10 to 0.31), or total bilirubin for progesterone receptor membrane component 1 (causal *β* = −0.20, 95% CI −0.26 to −0.12) and phosphodiesterase 3A (causal *β* = −0.13, 95% CI −0.18 to −0.08).

### Protein determinants help interpret disease signatures

To establish clinical and biological relevance of our findings, we first tested whether any of the protein targets were associated with the onset of 20 incident diseases in a randomly selected subcohort of the European Prospective Investigation into Cancer (EPIC) Norfolk Study (*N* = 875; Supplementary Tables 12 and 13). We identified a total of 304 protein-incident disease associations in survival analyses at a 5% FDR (268 positively associated, 36 inversely associated with risk of disease onset), comprising 271 unique protein targets and 19 diseases (Supplementary Table 14).

**Fig. 6:**
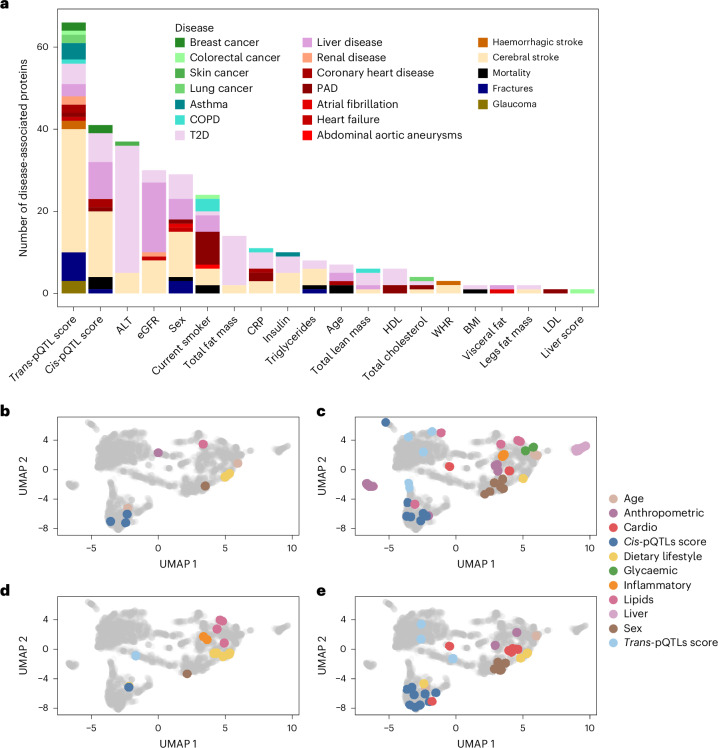
Annotation of incident disease-associated proteins by major and causal determinants. **a**, Disease-associated proteins per major biological contributor to the plasma proteome. **b**–**e**, All-cause mortality (**b**), T2D (**c**), PAD (**d**) and liver disease (**e**) associated proteins are annotated on the UMAP projection (Fig. 2b) by their major phenotypic or genetic contributor. WHR, waist-to-hip ratio; COPD, chronic obstructive pulmonary disease; T2D, type 2 diabetes; PAD, peripheral arterial disease.

Given the underlying structure of protein clustering by major biological factors associated seen in the UMAP, we mapped protein–disease associations on the UMAP to visualize potential enrichment of biological factors underlying these associations, for example, proteins associated with type 2 diabetes (T2D) were enriched in proteins strongly associated with total fat mass (OR 17.01, 95% CI 3.66 to 159.93); peripheral arterial disease (PAD) associations were enriched in proteins strongly associated with smoking status (OR 16.79, 95% CI 4.87 to 58.16) and high-density lipoprotein (HDL) cholesterol (OR 9.26, 95% CI 0.78 to 70.83) (Fig. 6a). The apparent enrichment of proteins mostly associated with the *trans*-pQTL score in those associated with cerebral stroke might result from spurious associations introduced by platelet activation, which affects both protein measurement and subclinical phenotypes associated with disease risk. All-cause mortality, chronic obstructive pulmonary disease (COPD) and liver disease associations were enriched in proteins strongly associated with age (OR 14.05, 95% CI 1.17 to 104.27), smoking status (OR 15.89, 95% CI 1.98 to 127.58) and kidney function (OR 18.44, 95% CI 6.95 to 52.29), respectively (Fig. 6b–e).

These findings clearly highlight the potential to refine known risk factors. For example, total fat mass is a major risk factor for T2D and potentially causally related to carboxypeptidase M (*β* = 0.04, 95% CI 0.02 to 0.06), which in turn was strongly associated with incident T2D (hazards ratio (HR) 2.04, 95% CI 1.54 to 2.67). Similarly, current smoking status is an associated causal determinant for alkaline phosphatase placental type (ALPP), PAD (HR 1.32, 95% CI 1.15 to 1.52) and COPD (HR 1.51, 95% CI 1.26 to 1.81). ALPP is expressed in lung epithelium^20^, suggesting smoking-induced damage to these tissues might result in increased leakage into the peripheral circulation, consistent with the MR causal estimates (*β* = 0.29, 95% CI 0.21 to 0.37). This protein might therefore represent a refined blood-based biomarker of lung damage among smokers. In total, 36.8% of disease-associated proteins (*N* = 112) were causally associated with a major modifiable determinant (Supplementary Table 14).

In contrast, around 39% of disease-associated proteins were only poorly explained (less than 10% of their variation in plasma) by genetic factors and participant characteristics investigated and thus might point to yet unrecognized pathways. For example, tenomodulin was strongly associated with PAD (HR 1.31, 95% CI 1.17 to 1.46), but despite our comprehensive assessment we were only able to explain 4.23% of variation in plasma levels, mainly explained by the *trans*-pQTL score (1.52%).

### Major determinants as modifiers of disease mediators

**Extended Data Fig. 3:**
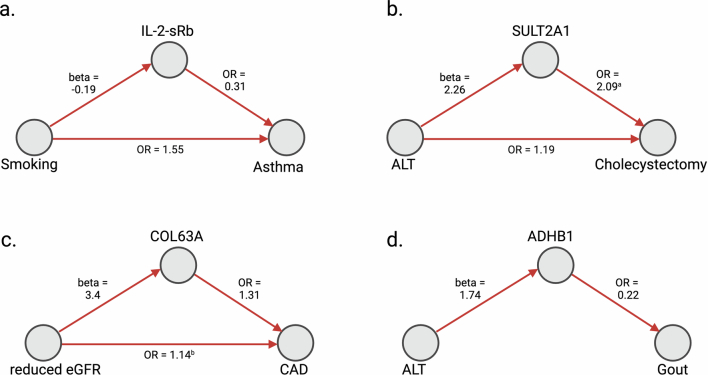
Examples of prioritization of putative risk factor-protein-disease causal triangles. **a-c**, Examples of potential molecular mediators of causal associations between risk factors and diseases. We report effect estimates from Mendelian Randomization (beta coefficients for risk factor to proteins, and OR for risk factor or proteins to diseases. ^a^Causal effects based on the Wald ratio estimate reported by Pietzner et al. ^b^Causal effect reported by Gaziano et al. in individuals with eGFR ≤ 60 mL/min/1.73m2. We note that in **c**, we present the inverse beta coefficient as we do this for reduced eGFR, rather than eGFR (as performed in the analysis) to orient the triangle to direction of increased risk. **d**, Alternative scenario representing independent effects converging on the same protein. This example might be best explained by the known modifying effect of the lead cis-pQTL for ADH1B (rs1229984) on alcohol consumption. Therefore, ADH1B might be independently associated with the risk of gout and ALT through alcohol consumption; and in turn, the observed MR finding between ALT and ADH1B plasma levels might rather reflect reverse causality. Created with BioRender.com.

Much interest has recently been focused on the identification of novel druggable targets in proteogenomic studies, largely neglecting the possibility to modify disease-causing proteins via alternative routes, including lifestyle interventions. We therefore sought to identify such modifiable phenotypic factors by systematically establishing suggestive causal triangles (Extended Data Fig. 3). To this end, we extracted a total of 42 likely causal protein–disease connections from our previous work^4^, which involved 31 phenotypes for 19 proteins that were causally associated with 9 modifiable determinants (Supplementary Table 15). Among these, we identified several examples that probably represent causal triangles (Extended Data Fig. 3a).

For example, we had previously identified a shared genetic signal (rs2284033, posterior probability of 98.96%)^4^ between interleukin-2 receptor subunit β (IL-2-sRβ) and asthma (causal OR 0.31, 95% CI 0.20 to 0.46). In turn, current smoking status was causally associated with IL-2-sRβ plasma levels (MR *β* = −0.18, 95% CI −0.26 to −0.10; tier 2 finding) (Supplementary Table 15), suggesting a possible biological mechanism for the effect of smoking cessation on reducing adult-onset asthma risk (causal OR 1.55, 95% CI 1.16 to 2.07).

Other examples highlighted the potential benefit from monitoring and early intervention, to delay or slow down progression of not yet reversible conditions. Impaired kidney function, and eventually chronic kidney diseases (CKD), is a strong an independent risk factor for coronary artery disease (CAD)^21^. We identified collagen type VI α 3 chain (COL6A3) as a potential mediator of this adverse process (Extended Data Fig. 3d). Even eGFR within the normal range explained a large amount of variation in plasma COL6A3 levels (10.5%), and MR supported this to be a potentially causal association (MR *β* estimate −3.47, 95% CI −4.06 to −2.86). Higher protein expression of COL6A3, in turn, was probably causal for a higher risk of CAD (rs1167793, posterior probability of 95.04%, causal OR 1.31, 95% CI 1.19 to 1.44). COL6A3, a component of type VI collagen that is found in most connective tissues is considered a marker of fibrosis^22^ and mutations in the protein coding gene have been described in patients with Bethlem myopathy and other rare neuromuscular diseases^23^, including reports on cardiac abnormalities^24^. Kidney fibrosis has been established as one of the main markers of progressive decrease in kidney function and a final common outcome of CKD^25^. Our findings therefore point to potential underlying mechanisms related to extracellular matrix remodelling and fibrotic processes^26^ linking these common comorbidities.

## Discussion

Profiling thousands of proteins from readily accessible biospecimens is becoming a standard tool in drug discovery and biomarker research, but we understand little about the individual determinants of protein abundance outside a cellular context that can also inform alternative treatment strategies to target disease-causing proteins. Here, we systematically identified modifiable factors associated with plasma protein abundance and provided evidence of causal associations between major determinants and a substantial fraction of the ‘modifiable’ proteome (~14%). We demonstrated how our results can inform biomarker discovery studies for incident diseases and provide novel insights into how established risk factors might eventually translate into disease onset. Most importantly, our results provide candidate molecular mediators through which tangible and safe interventions might modulate disease risk. We therefore contribute evidence to support the effectiveness of established interventions and provide candidate drug targets for examples in which there are currently no established effective behavioural modifiers.

Large-scale efforts have focused on characterizing the genetic determinants of plasma protein levels, to facilitate identification of candidate disease mediators and potential drug targets^3–5^. However, we demonstrated here that plasma levels of more than 30% of the protein targets are best explained by non-genetic factors and rather highlight systemic effects. These included liver injury and reduced kidney function as major determinants of increased plasma protein levels, as well as lifestyle factors (for example, smoking) or a low-grade inflammatory state, which shifts the composition of the plasma proteome. Underlying mechanisms may include cellular leakage of hepatocytes and other cells challenged by endogenous and exogenous stimuli (such as smoke-induced damage to lung epithelium leading to ALPP leakage), but also adaptive mechanisms such as increased cytokine signalling in response to a pro-inflammatory milieu triggered by increased activation of tissue resident macrophages in lung or adipose tissue^27^. In general, our results provide a comprehensive resource to enhance our understanding of what does and does not relate to plasma protein levels. For example, we did not observe much evidence that dietary patterns contribute to the plasma proteome, whereas they are highly associated with the circulating metabolome^28^.

We demonstrated the value of our findings to contextualize disease-associated proteins and the mechanisms these might relate to. While protein functions have been well characterized and can be readily explored with functional annotation tools, most of this information comes from cellular or mouse models, in which experimental conditions are carefully controlled to isolate the influence of specific perturbations. In contrast, epidemiological studies rely on free-living people that are subject to a range of exposures. We therefore provide a unique resource to better understand proteomic findings from the increasing number of observational association studies, specifically for clinical case–control studies of less common diseases for which detailed phenotyping is often hard to achieve. Our systematic approach further enabled identification of refined biomarkers of common risk factors, which might represent objective proxies for lifestyle factors, such as ALPP for smoking status, which are based on self-report and are therefore subject to high measurement error due to misreporting.

We provide evidence for modifiable factors that could modulate disease risk by targeting disease-causing proteins, providing an alternative to pharmaceutical interventions. Some of these examples included well-known relationships such as the increased risk of asthma in smokers^29^, for which we provide evidence of IL-2-sRβ as a potential mediator that could reduce the risk of asthma upon smoking cessation. While these findings provide a source of potential links between risk factors, molecular mediators and disease, we note that we could not fully establish the direction of causality in this setting, which is a limitation of our study. Therefore, these findings require further follow-up in clinical trials to establish causality.

A major strength of this study was the deep phenotyping done in the Fenland study, enabling a systematic characterization of the determinants of the plasma proteome that explained a substantial amount (≥10%) of variation in plasma levels for a third of assayed proteins. However, while we provide a comprehensive map of the potential associated factors and determinants of the plasma proteome, the exact biological mechanisms involved in regulating their levels remain to be established. We highlight that our findings need to be interpreted in relative terms, with the same change in a determining factor maybe leading to strong or mild changes in plasma protein levels depending on the level of homeostatic control. Furthermore, our results are dependent on the assumption of additive effects and considerably larger studies are needed to establish potential interaction effects among phenotypic characteristics associated with plasma protein levels. Our study included relatively healthy participants, which meant we were not able to study the effect of medications or prevalent diseases in great detail, even though we included genetic risk scores for several common diseases among the list of explanatory variables. Furthermore, the affinity-based technology used to measure the plasma proteome, relied on a conserved protein structure for accurate quantification, which might be affected by genetically induced isoforms that might well account for some of the very high amounts of explained variance by *cis*- and *trans*-pQTLs. A recent study using a mass spectrometry-based proteomic platform suggested up to one-third of pQTLs can represent false positives arising from these ‘epitope effects’^30^, which might lead to overestimation of the proportion of variance explained by the pQTL scores in our study. Furthermore, affinity-based proteomics cannot differentiate between protein isoforms that could be influenced by different biological factors. Future efforts should therefore focus on providing validation of the major determinants of the plasma proteome using an alternative, orthogonal proteomic technology in independent studies including participants of a wider age range and ethnic diversity. We also observed a strong impact of technical factors on many protein targets that might in part account for variable findings and poor replication across different studies. Technical variation will also affect the exposure side, and we cannot preclude that some of the estimates presented are affected by measurement error. Finally, while we provide selected examples for which risk factors may exert their effect via circulating proteins, we are currently missing large sets of genetic instruments for plasma proteins to assess these hypotheses formally.

We demonstrated how systematic identification of the major determinants of plasma protein abundance provides the much-needed insights into the biological drivers associated with shifts in the plasma proteome and potential actionable behavioural modification that could modulate disease mechanisms.

## Methods

### Study design and clinical assessment

The Fenland study is a population-based cohort of 12,435 men and women born between 1950 and 1975 who underwent detailed phenotyping at the baseline visit from 2005–2015. Ethical approvals were obtained from the Cambridge Regional Ethics Committee (ref. 04/Q0108/19) and all participants provided written informed consent. Participants were recruited from general practice surgeries in Cambridge, Ely and Wisbech (United Kingdom). Exclusion criteria of the Fenland study included pregnancy, prevalent diabetes, an inability to walk unaided, psychosis or terminal illness.

At the baseline assessment on phase 1 of the study, participants provided information on lifestyle and general health. Anthropometric and objective clinical measurements of physical activity, cardiorespiratory fitness and body composition were taken^31^. Blood samples were collected for further metabolic assessment. Glucose and insulin concentrations were measured at fasting and at 2 h post-glucose challenge. Lipid profiles (triglycerides, HDL cholesterol and total cholesterol), ALT, ALP, CRP and serum creatinine (assayed in a Dade Behring Dimension RxL analyser) were measured at fasting and HbA1c was also measured (Tosoh Bioscience, TOSOH G7 analyser). LDL cholesterol was calculated with the Friedwald formula^32^. BMI was calculated as weight (kg) divided by the square of height (m^2^). eGFR was calculated by the CKD-EPI equation using serum creatinine^33^.

Hepatic steatosis (‘liver score’) was evaluated by an abdominal ultrasound and images were scored by two trained operators. Criteria used for scoring included: increased echotexture of the liver parenchyma, decreased visualization of the intra-hepatic vasculature and attenuation of ultrasound beam. A normal liver was considered as a score from 3 to 4, mild steatosis from 5 to 7 and moderate steatosis from 8 to 10, and severe steatosis was ≥11 (ref. ^34^).

Participants completed DEXA scan measurements using a Lunar Prodigy advanced fan beam scanner (GE Healthcare) performed by trained operators using standard imaging, positioning protocols and manually processed according to a standardized procedure described previously^35^. Abdominal visceral and subcutaneous fat mass was estimated using the DEXA software.

Participants recorded the frequency and portions of consumption of foods and beverages by completing a validated 130-item, semi-quantitative food frequency questionnaire for the assessment of habitual diet^36^. Overall dietary quality was evaluated using an index of dietary accordance with the Dietary Approaches to Stop Hypertension (DASH) diet, adapted from that of Fung et al.^37^. The index consists of eight dietary components (grains/grain products, vegetables, fruits, low-fat/fat-free dairy, red and processed meat, nuts/seeds/dry beans, dietary sodium and foods high in added sugar).

A detailed description of objective physical activity assessment has been previously described^31^. Briefly, participants were fitted with a combined heart rate and uniaxial movement sensor (Actiheart, CamNtech) attached to the chest with standard electrocardogriam electrodes^38^. Participants had their heart rate measured continuously during a 6 min supine rest test and then underwent a submaximal treadmill test consisting of 9 min of walking on the flat surface with increasing speed, 6 min walking at increased gradient and 5 min of jogging on the flat surface^39^. Heart rate was measured continuously, and the test was terminated when heart rate reached 90% of age-predicted maximum, or had been above 80% for longer than 2 min, or the participant requested to stop. At the end of the clinical visit, participants were asked to wear the heart rate and movement sensor, initialized to collect data at 1 min resolution, for the following 6 days^38^, and to return the monitor by freepost. Heart rate was individually calibrated using parameters obtained from the treadmill test and combined with acceleration in a branched equation model to calculate instantaneous physical activity energy expenditure as previously described^31^. Data collection and analysis were not performed blinded as there were no experimental conditions.

### Genotyping and imputation

This study is based on the subset of unrelated individuals from the Fenland cohort (phase 1; Supplementary Table 1) with genotyping from the Affymetrix UK Biobank Axiom array (OMICs, *N* = 8350). No statistical methods were used to pre-determine sample sizes but our sample sizes are similar to those reported in previous publications^4,12^. Samples were excluded for the following reasons: (1) failed channel contrast (DishQC <0.82); (2) low call rate (<95%); (3) gender mismatch between reported and genetic sex; (4) heterozygosity outlier; (5) unusually high number of singleton genotypes; or (6) impossible identity-by-descent values (samples for which the proportion of the genome where both alleles are identical by descent was 10–23%, while the proportion of genome where one allele is identical by descent was zero). SNPs were removed if: (1) call rate was <95%; (2) clusters failed Affymetrix SNPolisher standard tests and thresholds; (3) multiple allele frequency (MAF) was significantly affected by plate; (4) SNP was a duplicate based on chromosome, position and alleles (selecting the best probeset according to Affymetrix SNPolisher); (5) Hardy–Weinberg equilibrium showed *P* < 10^−6^; (6) did not match the reference; or (7) there was an MAF of 0.

Autosomes were imputed to the Human Reference Consortium (r1) panel using IMPUTE4, and the X chromosome was imputed to HRC.r1.1 using the Sanger imputation server^40^. Imputation to the UK10K+1000Gphase3^41^ panel using the Sanger imputation server was also done to obtain additional variants that do not exist in the HRC reference panel. Variants with MAF <0.001, imputation quality (info) <0.4 or Hardy–Weinberg equilibrium *P* < 10^−7^ in any of the genotyping subsets were excluded from further analyses.

### Proteomic profiling

Proteomic profiling from fasted samples was done using the SomaScan v4 platform. The SomaScan platform uses modified single-stranded DNA sequences (aptamers or SOMAmer reagents) to target proteins. Concentration is approximated as relative fluorescence units (RFUs) using DNA microarrays. To account for variation in hybridization within runs, hybridization control probes are used to generate a hybridization scale factor for each sample. To control for total signal differences between samples due to variation in overall protein concentration or technical factors such as reagent concentration, pipetting or assay timing, a normalization procedure (adaptive median normalization) was applied to the data. This ensures quantitative comparability between sample sets. Briefly, for adaptive median normalization, a ratio between each aptamer’s measured value and a reference value from an external reference population is computed. The median of these ratios is computed for each of the three dilution sets (20%, 0.5% and 0.005%) and applied to each dilution set to shift the intrapersonal distribution of protein intensities accordingly to match the reference population. Samples are removed if they do not meet the standard acceptance criteria for scaling factors with values outside of the recommended range (0.25–4) or are flagged as technical failures. Detailed normalization, calibration data and quality control processes for SomaLogic’s assay have been previously described in detail^42^. At a protein level, only human protein targets are taken forwards for subsequent analysis. Aptamers’ target annotation and mapping to UniProt accession numbers as well as Entrez gene identifiers were provided by SomaLogic and we used those to obtain genomic positions of protein encoding genes. There were no missing values in the data. RFUs were inverse rank normalized for all downstream analyses. There were no ties in the data.

### Feature selection

We generated a comprehensive list of phenotypic, genetic and technical variables to investigate their contribution to the variance explained in plasma protein levels (Supplementary Table 2). We imputed missing values as implemented by the missForest R package^43^ that enabled handling mixed-type variables under a unified framework. We generated weighted genetic risk scores (GRS) using previously published genome-wide significant variants for each of the proteins (one *cis*-pQTL score using variants within 500 kb of the gene body and one *trans*-pQTL score using variants further away than those 500 kb)^4^, anthropometric^35,44^, glycaemic^45,46^ and cardiovascular traits^47,48^. These were generated by weighting the SNP dosage by the *β* coefficients or log(ORs). Finally, we selected a set of variables that might inform on technical variation and sample handling factors, including study test site, appointment year and season, and the first principal component from the proteins in each of the three dilutions sets, as implemented by the SomaScan v4 platform, as a proxy of sample handling. A complete list of the 60 variables that were included in this study are listed in Supplementary Table 2.

We implemented an initial feature selection step, to identify the most comprehensive (that is, least sparse) set of explanatory variables for each of the 4,979 target proteins, while avoiding inclusion of strongly correlated variables that would lead to biased estimates or model failure because of multicollinearity. We note that this framework might still lead to variables with some degree of correlation, or with strong biological relationships that may not translate into numerically strong correlations in the data. We divided all individuals into a training (75%) and testing set (25%). For each target protein, we ran least absolute shrinkage and selection operator (LASSO) regression over 100 subsamples of the training set (taking 80% each time) using the matrix of complete phenotypic, technical and genetic variables as an input. In addition to the explanatory variables, we included three random variables from a normal distribution in the feature selection procedure. In each iteration we ran repeated cross-validation (ten repeats of ten-fold), using a grid search to tune the hyperparameter lambda (Supplementary Fig. 1). This was implemented using the R packages caret^49^ and glmnet^50^. Explanatory variables and the three random variables were ranked according to the number of times these were selected in the final cross-validated model, that is, scores ranging between 0 and 100. We used rankings for the random variables to determine a threshold for selection of explanatory variables to be included in downstream analyses.

### Variance explained in plasma protein abundances

Variables ranked above the random variables were included into linear mixed models fitted on the entire set of individuals with the variancePartition R package^51^. We used this method to quantify variation in the plasma levels of each of the target proteins attributable to each of the explanatory phenotypic, technical and genetic variables selected to be important. Randomization was not necessary since our study’s aim was to identify and quantify the influence of biological and technical variables on plasma protein levels in the population. Categorical variables were modelled as random effects, while continuous variables were modelled as fixed effects. We note that we performed this variance decomposition analysis in the entire set of individuals as our aim was to quantify the individual contributions of explanatory variables under a joint model, rather than optimize a model to predict plasma protein levels, which would suffer from over-fitting from such as approach. However, we still compared the total proportion of variance explained (r^2^) from the linear mixed models with the r^2^ from the LASSO models applied only in the test set, showing good consistency (Pearson correlation *r* = 0.88), to ensure no strong selection biases introduced by the feature selection procedure performed in a subset of individuals (75%). We found good agreement between the R2 from each of these approaches (Pearson correlation *r* = 0.88). The relationship between the explanatory variable and the protein levels were assumed to be linear but this was not formally tested. We identified the major biological determinant for each target protein as the phenotypic or genetic variable that explained the largest proportion of variance in plasma levels of the corresponding protein.

We used UMAP to visualize any potential underlying structure in the plasma proteome according to factors contributing to the explained variation in plasma protein abundances. UMAP is a dimensionality reduction technique that enables better preservation of the data’s global structure compared with similar methods such as *t*-distributed stochastic neighbor embedding. UMAP is a dimensionality reduction technique that optimizes a low-dimensional graph to be as similar as possible to a high-dimensional graph representation of the data. We applied UMAP on the matrix of explained variation by all genetic factors and participant characteristics (after regressing out the contribution by technical factors) on all protein targets. We used default values for most parameters used by the algorithm in the umap R package. Custom configuration was done for the following parameters as follows: random_state (seed for random number gnerator) = 10, metric = ‘pearson’, n_epochs = 1,000, init = ‘random’. We further tested different values for the nearest neighbour parameter (values from 5 to 25). While there were no major differences, we determined by visual inspection that the clearest clustering was achieved by setting 13 nearest neighbours. Colouring of the UMAP plot was performed according to the major explanatory factor (Supplementary Table 5) for each of the proteins.

### Heritability estimates

We used genome-wide genotype data to determine SNP-based heritability for all protein targets with at least one pQTL (either in *cis* or *trans*). We generated a genetic relationship matrix using GCTA v.1.90 (ref. ^52^) from all variants with MAF >1% to calculate SNP-based heritability as implemented by biMM^53^.

### Comparison with the Olink platform

For a subset of 500 Fenland participants proteomic profiling was done in plasma samples using 12 Olink 92-protein panels. These participants were selected at random from the largest set of Fenland participants that has been genotyped with the same array, and attended the main study test site. Values below the limit of detection were included in the analyses. We excluded 15 samples that did not meet the quality threshold recommended by Olink, leaving 485 individuals for analysis^12^. For comparison, we restricted analyses to a set of 273 matching assay pairs (266 unique Olink assay targets), that were well correlated between the two assays (Spearman *r* > 0.6). We fit linear mixed models using the variancePartition R package^51^, including the same variables as those selected for the SomaScan analyses, except for study test site, as these 485 participants attended the same study test site. Furthermore, we included the first principal component from all Olink proteins, compared with proteomic principal components by dilution factor as derived for the SomaScan analyses.

### Tissue-specific, secretome and druggable annotation

We programmatically downloaded tissue expression data from the Human Protein Atlas (HPA) for all SomaScan v4 proteins in JSON format (on 22 March 2023). Fifteen proteins (PTCHD3, PNLIPRP2, CARD17, GAGE2D, GSTT1, CCL4L1, CCL3L1, AREGB, C5orf38, LILRA3, PQLC2L, C1orf186, TYMSOS, C7orf69 and KIR2DS4) assayed by the SomaScan v4 platform were not found on HPA.

We performed a two-sided Fisher’s exact test to determine whether tissue-specifically expressed proteins were enriched among those best explained by the same major biological factors. We defined tissue- or cell-type specific as ‘enhanced’, ‘enriched’ or ‘group enriched’ according to HPA classification. We further performed two-sided Fisher’s exact test to determine whether proteins predicted to be secreted according to the HPA annotation were significantly enriched among any of clusters of proteins best explained by the same biological influence.

### MR

We performed two-sample summary data MR analyses to assess whether major modifiable biological contributors were causally related to their corresponding protein targets. We identified genetic instruments for the modifiable determinants from publicly available genome-wide significant variants^35,44,47,54–67^ (Supplementary Table 10). We ensured independency of instruments by applying a clumping algorithm implemented in the TwoSampleMR R package^68^, to prune SNPs in linkage disequilibrium (LD) using a threshold of *r*^2^ = 0.01. For specific variants that were not present in the protein genome-wide association studies, LD proxies were searched for with the default TwoSampleMR parameters (using data from the 1,000 genomes and a *r*^2^ threshold of 0.8). We used radial regression inverse variance-weighted (IVW) method as the main analysis, using exact weights. This method more accurately quantifies heterogeneity and enables identification of outlier instruments, determined by their contribution to global heterogeneity, quantified by Cochran’s *Q*-statistic^69^, and exact weighting estimates are much less affected by regression dilution bias. The main rationale to use radial MR as the major analysis instead of classical IVW analysis was to exclude genetic instruments mapping to the regions encoding the protein being used as an outcome, but that are unrelated to the often very strong *cis*-pQTL. The radial MR framework is likely to flag these instruments as outliers given their disproportionately large effect on the protein compared with the exposure. If these instruments were included, these could introduce evidence for reverse causality, that is, in the direction of the protein to the risk factor. For example, we showed previously that polygenic scores for lung function were strongly associated with plasma levels of QSOX2, which completely vanished once accounting for a genetic signal in moderate LD between one of the lung function instruments and the corresponding *cis*-pQTL^13^. We excluded instruments deemed as outliers using a *P*-value threshold of 0.05. We report IVW estimates under a random-effects model, which enables adjusting inferences about the causal effect to take into account the additional uncertainty that is present when there is heterogeneity due to pleiotropy. In practice, substantial heterogeneity is generally present in MR studies for common risk factors and traits, and therefore, a random-effects model is better suited than a fixed effects model for this systematic analysis. We consider causal determinants with evidence of a significant causal association at a 5% FDR threshold (for the number of proteins and determinants tested), but we report the raw *P* value in the main text. ORs represent the change in odds per 1 s.d. increase in genetically predicted protein levels. We used the RadialMR R package to implement these analyses^69^. We performed additional sensitivity analyses using three different MR methods, including classical IVW^70^ analysis, MR-Egger^71^ and weighted median^72^, to categorize findings in two tiers according to the level of evidence obtained. Tier 1 findings included putative causal associations from the main analysis, which were directionally consistent, at least nominally significant in all analyses and showed no evidence of pleiotropy, that is, the Egger intercept *P* value >0.05, whereas tier 2 included the remainder of putative causal associations from the main analysis. We provide a completed STROBE-MR checklist as Supplementary Table 16.

We prioritized putative risk-factor–disease triangles by cross-referencing proteins causally determined by a risk factor and protein–disease edges from our previously published proteogenomic phenome-wide colocalization map based on *cis*-pQTL regions^4^. We restricted this look-up to binary endpoints, for which the lead pQTL reached at least genome-wide significance for the corresponding protein (*P* < 5 × 10^−8^).

We further reported the IVW MR estimates for the associations between genetically predicted modifiable determinants discussed and the disease risk. These analyses were performed using MR-Base to test for a causal association between smoking status and asthma and ALT and cholecystectomy using the summary statistic from the largest available genome-wide association studies. We further report causal estimates from previous MR studies, such as for the effect of eGFR on CAD and SULT2A1 on cholecystectomy.

### Survival analyses

We tested the association of protein plasma abundances with the 20 incident diseases and all-cause mortality with at least 15 incident cases in a random subcohort from the EPIC-Norfolk study (*N* = 875; Supplementary Tables 12 and 13; approved by the Norfolk Research Ethics Committee, ref. 05/Q0101/191), in which proteomic profiling was done at health check 1 using the SomaScan v4 platform. Normalization was performed by SomaLogic using adaptive normalization by maximum likelihood (ANML).

Hospitalization data, death registry and cancer registry records were obtained using National Health Service numbers through linkage with National Health Service digital. Vital status was ascertained for the entire EPIC-Norfolk cohort and death certificates were coded by trained nosologists according to the International Statistical Classification of Diseases and Related Health Problems, 10th Revision (ICD-10). For disease definitions, participants were identified as having an event if the corresponding ICD-10 code was registered on the death certificate (as the underlying cause of death or as a contributing factor) as the cause of hospitalization or recorded in the cancer registry. Since the long-term follow-up of EPIC-Norfolk comprised the ICD-9 and ICD-10 coding system, codes were consolidated. The current study is based on follow-up up to 31 March 2016. We used Cox proportional hazards models, adjusting for age at baseline and sex. Prevalent cases and incident cases within the first 6 months of follow-up were excluded from the analyses. Associations were deemed significant at an 5% FDR, but we report raw *P* values in the main text. We further computed incident disease associations using raw RFUs, that is, before ANML normalization, and only retained protein disease at 5% FDR from the ANML analyses that were also directionally consistent and at least nominally significant in the raw analysis. This is because while ANML removes the most likely spurious phenotypic associations from the data, care must be taken if the scaling factors are associated the outcome tested. Therefore, we applied this filter to remove protein–disease associations that may result from biases introduced by the ANML procedure.

We performed a two-sided Fisher’s exact test to determine whether disease-associated proteins were enriched in those best explained by specific major biological factors (per disease per major explanatory factor).

### Statistical analyses

Statistical analyses are described in detail throughout each of the above sections.

### Reporting summary

Further information on research design is available in the Nature Portfolio Reporting Summary linked to this article.

## Supplementary information

Supplementary Information Supplementary Fig. 1.

Reporting Summary

Supplementary Tables Supplementary Tables 1–16.

## Data Availability

Deposition of individual-level data in public repositories is not possible due to the ethical approval for these studies. However, data access for the Fenland and EPIC studies can be requested by bona fide researchers for specified scientific purposes through a simple application process via the study websites below. Data will either be shared through an institutional data sharing agreement or arrangements will be made for analyses to be conducted remotely without the necessity for data transfer; Fenland: https://www.mrc-epid.cam.ac.uk/research/studies/fenland/information-for-researchers and EPIC-Norfolk: https://www.mrc-epid.cam.ac.uk/research/studies/epic-norfolk. To accelerate the use of our results we generated an interactive webserver to query our data for all proteins and all explanatory variables tested (https://omicscience.org/apps/protdeterminants). Underlying data for all figures are available in the supplementary tables. Data from the Human Protein Atlas are publicly available (https://www.proteinatlas.org/).

## Code availability

Associated code and scripts for the analysis can be found via GitHub at https://github.com/comp-med/proteome-determinants-somalogic.git.
