# Supplementary material for: Mapping biological influences on the human plasma proteome beyond the genome
Source: Nat Metab. 2024 Sep 26;6(10):2010–23. doi: 10.1038/s42255-024-01133-5 (PMC11496106; doi:10.1038/s42255-024-01133-5)
Supplement: Supplementary file 1 — Supplementary Fig. 1. [file 42255_2024_1133_MOESM1_ESM.pdf]

nature metabolism

Supplementary information

https://doi.org/10.1038/s42255-024-01133-5

Mapping biological influences on the human plasma proteome beyond the genome

In the format provided by the authors and unedited

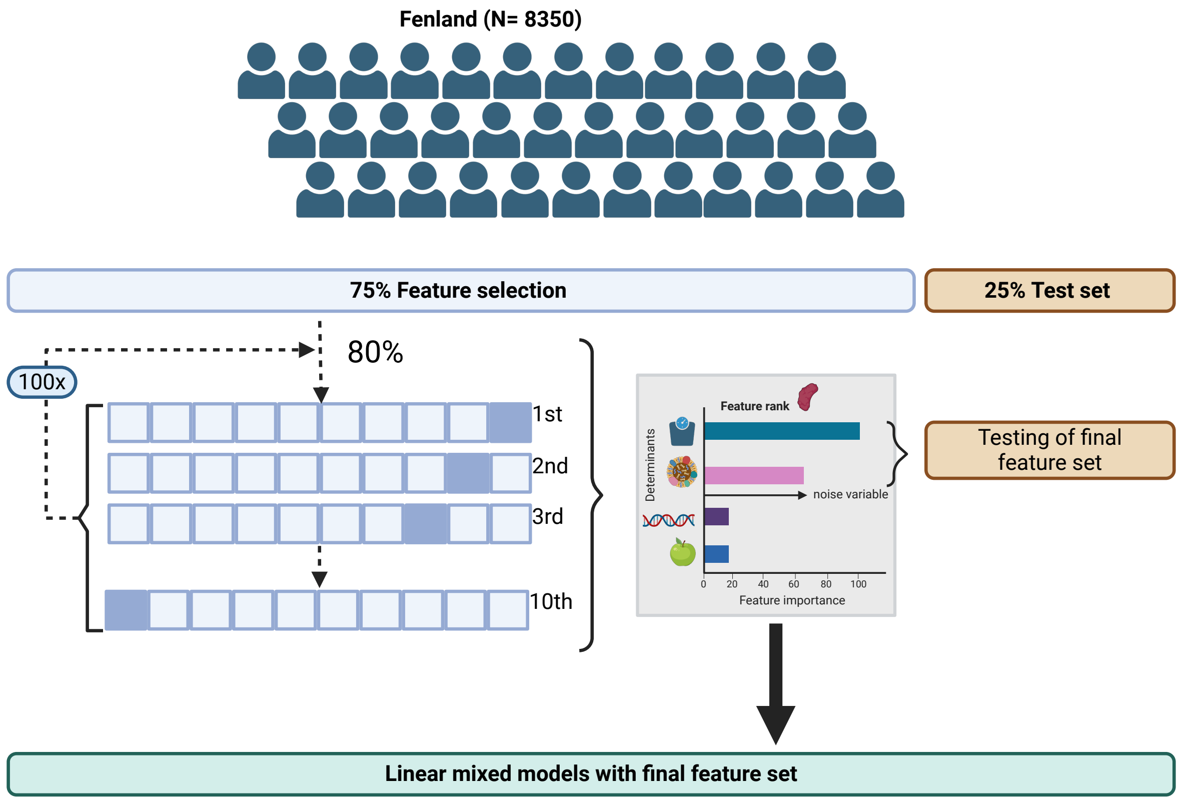

Supplementary Figure 1. General framework for feature selection and variance explained models. We divided all individuals into a training (75%) and testing set (25%). For each target protein, we performed feature selection, by LASSO over 100 subsamples of the training set (taking 80% each time) using the matrix of complete phenotypic, technical, and genetic variables as an input. In addition to the explanatory variables, we included three random variables from a normal distribution in the feature selection procedure. In each iteration we ran repeated cross-validation (10 repeats of 10 folds). Features ranked above random variables ('noise variables') were then tested in the test set. Separately, we used features ranked above random variable to run linear mixed models for each proteins to assess the proportion of variance attributable to each variable in all individuals. Figure created with BioRender.
