# Supplementary material for: Mapping biological influences on the human plasma proteome beyond the genome
Source: Nat Metab. 2024 Sep 26;6(10):2010–23. doi: 10.1038/s42255-024-01133-5 (PMC11496106; doi:10.1038/s42255-024-01133-5)
Supplement: Supplementary file 2 — Reporting Summary [file 42255_2024_1133_MOESM2_ESM.pdf]

nature portfolio

Corresponding author(s): Clauda Langenberg

Last updated by author(s): Jul 26, 2024

Reporting Summary

Nature Portfolio wishes to improve the reproducibility of the work that we publish. This form provides structure for consistency and transparency in reporting. For further information on Nature Portfolio policies, see our Editorial Policies and the Editorial Policy Checklist.

Statistics

For all statistical analyses, confirm that the following items are present in the figure legend, table legend, main text, or Methods section.

n/a	Confirmed	
☐	☒	The exact sample size (*n*) for each experimental group/condition, given as a discrete number and unit of measurement
☐	☒	A statement on whether measurements were taken from distinct samples or whether the same sample was measured repeatedly
☐	☒	The statistical test(s) used AND whether they are one- or two-sided<br>*Only common tests should be described solely by name; describe more complex techniques in the Methods section.*
☐	☒	A description of all covariates tested
☐	☒	A description of any assumptions or corrections, such as tests of normality and adjustment for multiple comparisons
☐	☒	A full description of the statistical parameters including central tendency (e.g. means) or other basic estimates (e.g. regression coefficient) AND variation (e.g. standard deviation) or associated estimates of uncertainty (e.g. confidence intervals)
☐	☒	For null hypothesis testing, the test statistic (e.g. *F*, *t*, *r*) with confidence intervals, effect sizes, degrees of freedom and *P* value noted<br>*Give P values as exact values whenever suitable.*
☒	☐	For Bayesian analysis, information on the choice of priors and Markov chain Monte Carlo settings
☒	☐	For hierarchical and complex designs, identification of the appropriate level for tests and full reporting of outcomes
☐	☒	Estimates of effect sizes (e.g. Cohen's *d*, Pearson's *r*), indicating how they were calculated

Our web collection on statistics for biologists contains articles on many of the points above.

Software and code

Policy information about availability of computer code

Data collection	No software was used for data collection.
Data analysis	Software: Lunar Prodigy advanced fan beam scanner (GE Healthcare). IMPUTE4; R (v4.1.2); GCTA (v.1.90)<br>R packages: variancePartition (v1.14.1), missForest (v1.5), glmnet (v.4.1), caret (v6.0), umap (v1.0), biMM (v1.0.0), TwoSampleMR (v.2.1), radial MR (v1.0), survival (v3.2-13). Code used for this study is found in the following GitHub repository: https://github.com/comp-med/proteome-determinants-somalogic.git

For manuscripts utilizing custom algorithms or software that are central to the research but not yet described in published literature, software must be made available to editors and reviewers. We strongly encourage code deposition in a community repository (e.g. GitHub). See the Nature Portfolio guidelines for submitting code & software for further information.

Data

Policy information about availability of data

All manuscripts must include a data availability statement. This statement should provide the following information, where applicable:

- Accession codes, unique identifiers, or web links for publicly available datasets
- A description of any restrictions on data availability
- For clinical datasets or third party data, please ensure that the statement adheres to our policy

Deposition of individual level data in public repositories is not possible due to the ethical approval for these studies. However, data access for the Fenland and EPIC

studies can be requested by bona fide researchers for specified scientific purposes through a simple application process via the study websites below. Data will either be shared through an institutional data sharing agreement or arrangements will be made for analyses to be conducted remotely without the necessity for data transfer. Fenland: https://www.mrc-epid.cam.ac.uk/research/studies/fenland/information-for-researchers. EPIC-Norfolk: https://www.mrc-epid.cam.ac.uk/research/studies/epic-norfolk.To accelerate the use of our results we generated an interactive webserver to query our data for all proteins and all explanatory variables tested (https://omicscience.org/apps/protdeterminants). Underlying data for all figures is available in the supplementary tables. Data from the Human Protein Atlas is publicly available (https://www.proteinatlas.org/).

Research involving human participants, their data, or biological material

Policy information about studies with human participants or human data. See also policy information about sex, gender (identity/presentation), and sexual orientation and race, ethnicity and racism.

Reporting on sex and gender	We included a total of 4404 and 3,946 participants who self-identified as women and men, respectively, and had matching karyotypes in our study. We systematically tested for an effect of sex on all protein levels.
Reporting on race, ethnicity, or other socially relevant groupings	We restricted our analysis to participants of white-European ancestry due to the very low number of people of non-European ancestry. We did not apply any other exclusion criteria with respect to race, ethnicity, or socially relevant groupings.
Population characteristics	Detailed characteristics can be found in Supplemental Table 1.
Recruitment	Participants were recruited from general practice surgeries in Cambridge, Ely and Wisbech (UK). Exclusion criteria of the Fenland study included pregnancy, prevalent diabetes, an inability to walk unaided, psychosis, or terminal illness.
Ethics oversight	Ethical approvals were obtained from the Cambridge Regional Ethics Committee (Ref 04/Q0108/19), and all participants provided written informed consent.

Note that full information on the approval of the study protocol must also be provided in the manuscript.

Field-specific reporting

Please select the one below that is the best fit for your research. If you are not sure, read the appropriate sections before making your selection.

☒ Life sciences ☐ Behavioural & social sciences ☐ Ecological, evolutionary & environmental sciences

For a reference copy of the document with all sections, see nature.com/documents/nr-reporting-summary-flat.pdf

Life sciences study design

All studies must disclose on these points even when the disclosure is negative.

Sample size	We used individual level data from 8350 participants of the Fenland cohort with available genotype and proteomic data.
Data exclusions	We excluded participants if they had missing genotype or proteomic data, as well as if they were not part of the largest genotyping effort done in the Fenland cohort.
Replication	No other study currently provides the depth and breadth of phenotypic and proteomic data for replication.
Randomization	N/A - Randomisation was not necessary since our study's aim was to identify and quantify the influence of biological and technical variables on plasma protein levels in the population.
Blinding	N/A - Blinding was no necessary since there was no randomisation nor experimental groups.

Reporting for specific materials, systems and methods

We require information from authors about some types of materials, experimental systems and methods used in many studies. Here, indicate whether each material, system or method listed is relevant to your study. If you are not sure if a list item applies to your research, read the appropriate section before selecting a response.

Materials & experimental systems

n/a	Involved in the study
☒	☐ Antibodies
☒	☐ Eukaryotic cell lines
☒	☐ Palaeontology and archaeology
☒	☐ Animals and other organisms
☒	☐ Clinical data
☒	☐ Dual use research of concern
☒	☐ Plants

Methods

n/a	Involved in the study
☒	☐ ChIP-seq
☒	☐ Flow cytometry
☒	☐ MRI-based neuroimaging
